# Role of Estrogen Signaling in Notch Pathway Activation in Sertoli Cells

**DOI:** 10.1007/s43032-025-01901-y

**Published:** 2025-06-12

**Authors:** Sylwia Lustofin, Alicja Kamińska, Barbara Bilińska, Julia Wajda, Anna Hejmej

**Affiliations:** Department of Endocrinology, Institute of Zoology and Biomedical Research, Faculty of Biology, Jagiellonian University, Gronostajowa 9, Krakow, 30-387 Poland

**Keywords:** 17β-estradiol, ESR1, ESR2, GPER1, Testis, Sertoli cell, Notch pathway

## Abstract

Sertoli cells constitute a somatic component of seminiferous epithelium in mammalian testis which supports the process of spermatogenesis. These cells are sensitive not only to the male sex hormones androgens, but also to their metabolites, estrogens. In this study, we aimed to explore the action of estrogen signaling in rodent Sertoli cells on the activity of canonical Notch pathway, which provides contact-dependent communication between the cells of seminiferous epithelium. Experiments performed on Sertoli cell line and primary Sertoli cell cultures demonstrated that 17β-estradiol, acting through estrogen receptor 2, enhances Notch1 activation, transcriptional activity of recombination signal binding protein for immunoglobulin kappa J region (RBPJ), interaction of RBPJ with *Hes1* promoter, and the expression of the *Hes1* effector gene and protein. On the other hand, 17β-estradiol by binding to estrogen receptor 1 reduces the expression of the *Hey1* effector gene. In contrast to nuclear estrogen receptors, no evident role of G protein-coupled estrogen receptor 1 in regulating Notch1 signaling activity in Sertoli cells has been found. The data presented indicate the role of nuclear estrogen receptor signaling in the control of Notch1 pathway activity in Sertoli cells, and thus reveal new mechanism of estrogen action in maintaining homeostasis of the seminiferous epithelium.

**Supplementary Information:**

The online version contains supplementary material available at 10.1007/s43032-025-01901-y.

## Introduction

The presence of endogenous estrogens in men and males of other mammalian species is well established and the role of these hormones has been of interest to researchers for several decades (for review see: [1]). Testicular estrogens, including 17β-estradiol (E2), act as hormones, but also as paracrine/autocrine factors activating estrogen receptors (ERs): nuclear estrogen receptor 1 (ESR1), nuclear estrogen receptor 2 (ESR2), and transmembrane G protein-coupled estrogen receptor 1 (GPER1), that are widely expressed in gonadal cells [2]. All three receptors are detected in somatic cells of seminiferous epithelium, called Sertoli cells (SC) [3]. Available data provide evidence for a role of a E2 in SC proliferation, differentiation, and survival, cellular energy metabolism and maintaining ion homeostasis as well as SC – germ cell adhesion, and sperm release [4–9]. Nevertheless, molecular pathways controlled by estrogen signaling in SC are still scarce understood.

The Notch pathway is a mechanism providing contact-dependent communication between neighboring cells, such as germ cells and SC during spermatogenesis. In the canonical pathway, the Notch1 receptor present in the membrane of SC is activated by transmembrane ligand of Delta-Serrate-Lag2 family expressed in the germ cell [10]. This induces the release of the Notch1 intracellular domain (N1ICD), that is next translocated to the nucleus and associates with recombination signal binding protein for immunoglobulin kappa J region (RBPJ) transcription factor [11]. The best characterized effectors of Notch pathway in SC are transcription factors with a basic helix-loop-helix structure (bHLH) belonging to hairy and enhancer of split family (Hes) and Hes-related family bHLH transcription factor with YRPW motif (Hey) [10, 12, 13]. Proper activity of Notch pathway is important for the course of spermatogenesis [14]. In SC Notch1-HES1/HEY1 signaling controls the production of glial cell-derived neurotrophic factor and retinoic acid-metabolizing cytochrome which are crucial for maintaining the spermatogonial stem cell niche [10]. Recent findings indicate that Notch pathway regulates also SC proteins engaged in blood‒testis barrier, spermatocyte apoptosis, and spermatid differentiation, as well as SC glucose metabolism and lactate production [13, 15, 16].

As documented in our recent studies Notch signaling activity in SC is modulated by testosterone and follicle-stimulating hormone [17–19], while the role of other hormones or paracrine factors has not been studied in detail. Importantly, in recent years the significance of interplay between estrogens and Notch-mediated communication has been identified in breast cancer cells as well as in cardiovascular system (for review see [20, 21]).

Therefore, to gain deeper insight into molecular mechanisms key for seminiferous epithelium homeostasis we aimed to explore the effects of estrogen signaling manipulation in rodent SC on Notch pathway activity and regulation of its target genes.

## Material and methods

### Cell culture and experiments

TM4 mouse SC line (Cat no. CRL-1715; ATCC, Manassas, VA, USA) was maintained under conditions recommended by the manufacturer. Primary SC were isolated from 20-day-old rat testes using enzymatic protocol and cultured as described previously [17, 22, 23]. Rat testes collection was carried out in accordance with EU Directive 2010/63 for the protection of animals used for scientific purposes and Polish legal requirements. Before experiments cells were serum starved for 24 h. Cells were incubated with: 1 nM E2 (Cat no. E2758; Sigma-Aldrich, St. Louis, MO, USA), 1 µM fulvestrant (ESR1/2 antagonist, Cat no. 5.31042; Sigma-Aldrich) or 10 nM G15 (GPER1 antagonist, Cat no. 3678, Tocris Bioscience, Minneapolis, MN, USA) alone or with 1 nM E2 or in the presence of 0.01% DMSO (a vehicle) for 24 h. For estrogen receptor knockdown, TM4 cells were transfected with Silencer Select siRNAs (assay ID: s65686 for ESR1; assay ID: s65689 for ESR2; assay ID: s94713 for GPER1, Thermo Fisher Scientific, Rocheford, Il, USA) using Lipofectamine RNAiMAX (Thermo Fisher Scientific) in serum-free Opti-MEM (Life Technologies, Gaithersburg, MD, USA), according to the manufacturer’s instructions. After 24 h cells were washed and 1 nM E2 or a vehicle was added for next 24 h. At least three independent experiments were performed, each in triplicate.

### Luciferase reporter assay

RBPJ reporter assay was performed using Cignal RBP-J Pathway Reporter Assay (Cat no. 336841; Qiagen, Hilden, Germany) according to the manufacturer’s instruction. In the first experiment TM4 cells were transfected with RBPJ reporter. Next E2, fulvestrant, or G15 was added after 24 h. In the second experiment, cells were transfected with RBPJ reporter and estrogen receptor specific siRNAs (listed above) followed by E2 or vehicle for 16 h. The luminescent activities of firefly and renilla luciferases were measured after 24 h using the Dual-Luciferase Reporter Gene Assay Kit (Promega, Madison, WI, USA). The firefly luciferase activities were normalized to the renilla luciferase activity.

### Real-time quantitative RT-PCR

Total RNA was extracted with Trizol reagent (Life Technologies), followed by DNA removal with TURBO DNA-free Kit (Cat no. AM1907; Ambion, Austin, TX, USA). Reverse transcription was performed with High-Capacity cDNA Reverse Transcription Kit (Cat no. 4368814; Applied Biosystems, Carlsbad, CA, USA). Next, StepOne Real-time PCR system (Applied Biosystems) was used for qPCR as described in detail [19]. Primer sequences are listed in Supplementary Table S1. Mean expression of *Actb, Hprt1,* and *Rpl13a* served as a reference for normalization. The relative mRNA expression levels were evaluated based on the 2^−ΔΔCt^ method.

### Immunoblotting

Samples were prepared and resolved by SDS-PAGE under reducing conditions, transferred to polyvinylidene difluoride membranes, and immunoblotted with the primary antibodies (Supplementary Table S2), as previously described in detail [19]. All immunoblots were stripped and reprobed with an anti-ACTB antibody. ImageLab software (Bio–Rad Labs., München, Germany) was used for densitometry. Each data point was normalized against its corresponding ACTB data point.

### Chromatin immunoprecipitation (ChIP)-qPCR assay

Chromatin of TM4 cells (incubated with 1 nM E2 or a vehicle for 24 h) was cross-linked using 1% formaldehyde. ChIP was performed with EZ-Magna ChIP Kit (Cat no. 17–10,086; Sigma-Aldrich) according to the manufacturer’s instructions. The following antibodies (Sigma-Aldrich): anti-RBPJ (10 μg/ml, Cat no. AB5790); anti-IgG (2 μg/ml; Cat no.12–371; negative control), anti-RNA polymerase II (2 μg/ml; Cat no. 05–623; positive control) were used. After DNA purification (QIAquick DNA Purification Kit, Qiagen) qPCR was performed as described in Sect. 2.4. The sequences of primers are: forward – GCGGCGGCAATAAAACATCC, reversed—AGCTGCAGTTTGACATCAGC for *Hes1* promoter; forward – AAAACAAGTGCTCCCCTTCC, reversed—CATGCAGCCAGACTCGTTTC for *Hey1* promoter [24, 25].

### Immunofluorescence

Immunofluorescence was performed on SC seeded on coverslips, according to previously described protocol [13]. The primary antibodies (Supplementary Table S2) were incubated overnight at 4 °C, followed by Cy3-conjugated anti-rabbit IgG (1:200; Cat no. A10520; Thermo Fischer Scientific) applied for 60 min. The cell nuclei were counterstained with 4′,6-Diamidine-2′-phenylindole dihydrochloride (DAPI). No background fluorescence was observed in the negative controls (not shown).

### Statistical analysis

Statistical differences in relative mRNA and protein expression were assessed using one-way ANOVA, followed by Tukey’s post hoc test or U Mann–Whitney test (Statistica 10, StatSoft Inc., Tulsa, OK, USA). Data are presented as means ± SD*.* Data are considered statistically significant at *p < 0.05, **p < 0.01, ***p < 0.001.

## Results

**Fig. 1:**
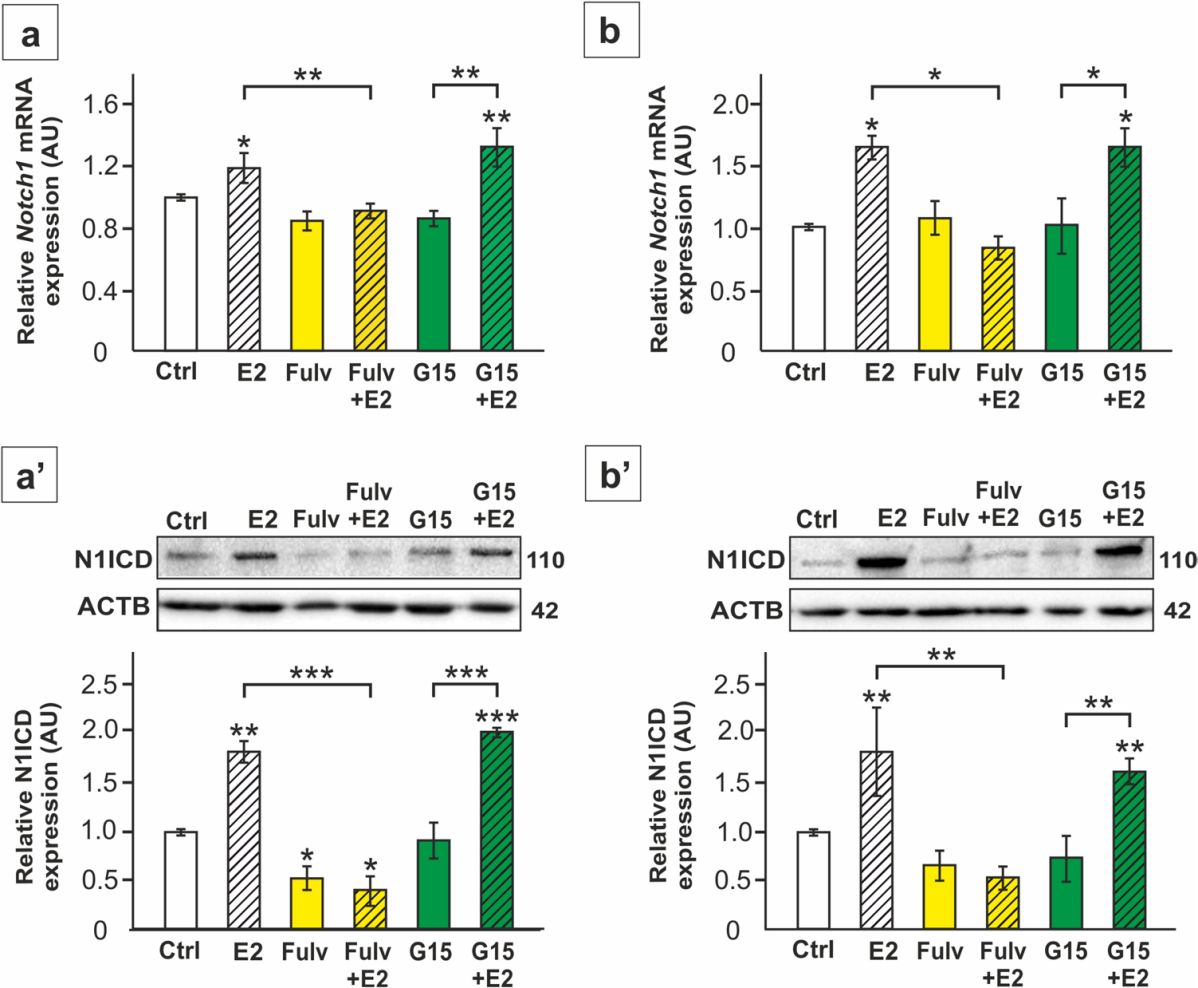
The effect of 17β-estradiol (E2) and antagonists of the estrogen receptors on *Notch1* mRNA and N1ICD protein expression in TM4 Sertoli cell line (**a**-**a’**) and primary rat Sertoli cells (**b**-**b’**). Cells were treated with 1 nM E2 and/or 1 µM fulvestrant (Fulv) or 10 nM G15, or a vehicle (Ctrl) for 24 h. **a**, **b** Relative expression of *Notch1* mRNA (RQ) following E2, Fulv or G15 was determined using RT-qPCR. The expression values of the individual genes were normalized to the mean expression of the reference genes (*Actb*, *Hprt1*, and *Rpl13a*) as an internal control. **a’**,**b’** Relative expression of N1ICD was determined with immunoblotting. Protein molecular weight (in kDa) is indicated. The protein levels (normalized to ACTB) within the control group were set at 1. The histograms are the quantitative representation of data (mean ± SD); *p < 0.05, **p < 0.01, ***p < 0.001

To investigate the effects of estrogens and the role of nuclear ERs and GPER1 in regulating Notch signaling, SC were treated with E2 in the presence or absence of specific antagonists of these receptors (fulvestrant or G15). Exposure of both primary and TM4 cells to E2 upregulated the expression of *Notch1* gene and increased the level of N1ICD protein (*p* < 0.05; p < 0.01) (Fig. 1a-b’). The effect of E2 was not detected in fulvestrant-treated cells, which suggests a role of ESR1 and/or ESR2 in mediating the action of E2 on Notch1/N1ICD. In contrast, incubation of SC with G15 did not prevent E2-stimulated expression of this receptor (*p* < 0.05; p < 0.01; p < 0.001), which implies a lack or a minor role of GPER1 in the effect of estrogens.

**Fig. 2:**
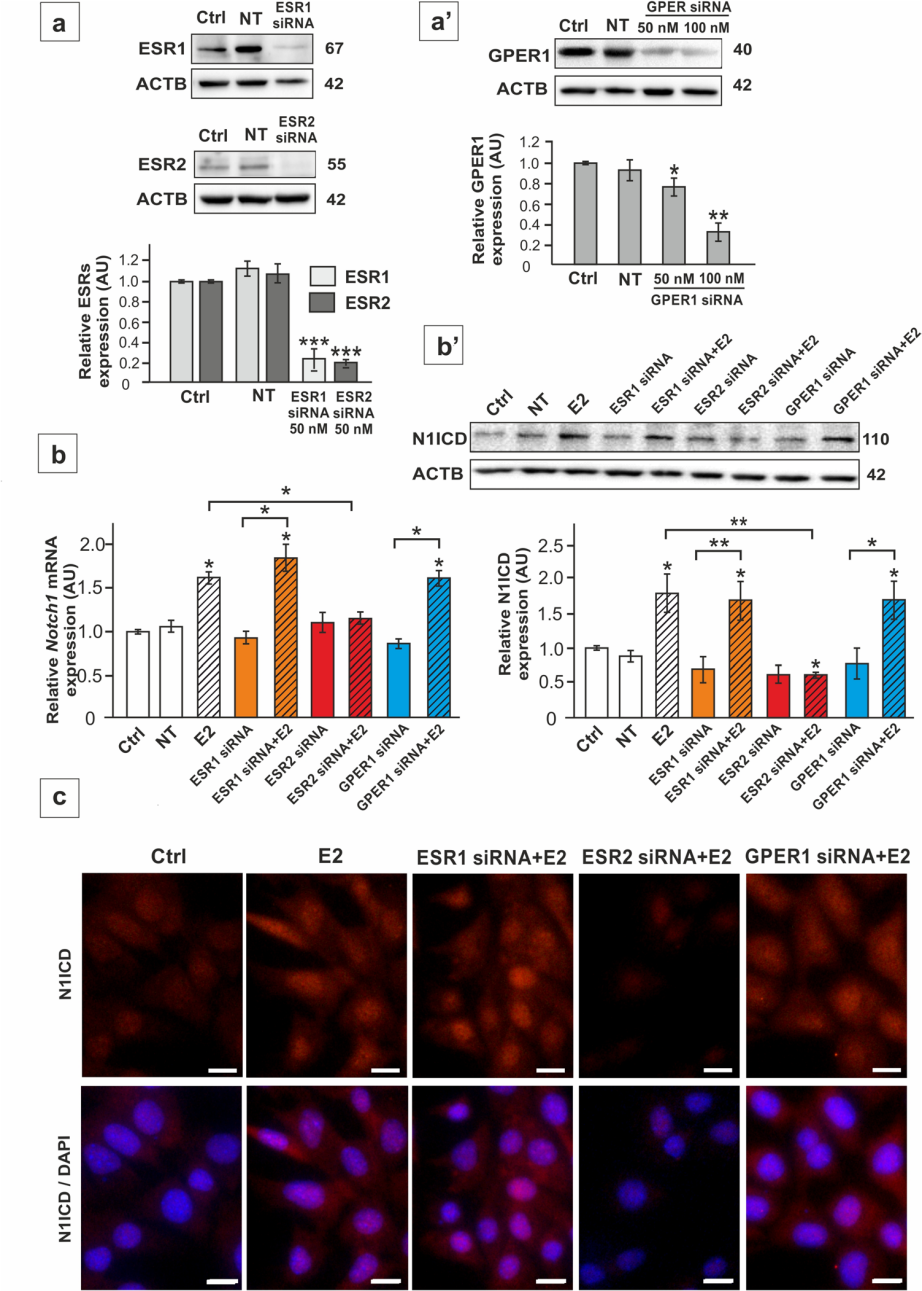
The effect of estrogen receptor silencing on ESR1, ESR2, and GPER1 protein expression (**a**, **a’**), *Notch1* mRNA (**b**) and N1ICD protein expression (**b’**- **c**) in TM4 Sertoli cell line. Cells were treated with Lipofectamine + non-targeting siRNA (NT), Lipofectamine + 50 nM ESR1 siRNA or 50 nM ESR2 siRNA or 50–100 nM GPER1 siRNA and 1 nM E2 or vehicle. **a**-**a’** Confirmation of silencing effectiveness of ESR1, ESR2, and GPER1 with immunoblotting. Protein molecular weight (in kDa) is indicated. The protein levels (normalized to ACTB) within the control group were set at 1. The histograms are the quantitative representation of data (mean ± SD); *p < 0.05, **p < 0.01, ***p < 0.001. **b** Relative expression of *Notch1* mRNA (RQ) following estrogen receptor silencing was determined using RT-qPCR. The expression values of the individual genes were normalized to the mean expression of the reference genes (*Actb*, *Hprt1*, and *Rpl13a*) as an internal control. **b’** Relative expression of N1ICD was determined with immunoblotting. Protein molecular weight (in kDa) is indicated. The protein level within the control group were set at 1. The histograms are the quantitative representation of data (mean ± SD); *p < 0.05, **p < 0.01, ***p < 0.001. **c** Immunofluorescence analysis of N1ICD in TM4 cells following estrogen receptor silencing. Irrespectively of treatment group, N1ICD signal (red) is localized predominantly to the nuclei. An increase in signal intensity is visible after E2, except for the *Esr2* knockdown group. Sertoli cell nuclei were stained with DAPI (blue). Lower panel represents the merged images. Scale bar = 10 µm

To confirm these findings, the expression of *Esr1*, *Esr2*, and *Gper1* was knockdown in TM4 cells prior to E2 treatment. The specific siRNAs downregulated the expression of the receptors by 60–80% (*p* < 0.05; p < 0.01; p < 0.001) (Fig. 2a-a'). Silencing of *Esr1* or *Gper1* had no effect on E2-stimulated *Notch1*/N1ICD expression, while *Esr2* knockdown clearly abolished the effect of E2 (*p* < 0.05; p < 0.01) (Fig. 2b-b'). Immunofluorescence analysis demonstrated an increase of N1ICD in the nuclei of E2-treated SC, indicating enhanced representation of the active form of the receptor. Immunofluorescence signal was decreased only in *Esr2* knockdown cells following E2 treatment (Fig. 2c). This is in agreement with immunoblotting results, identifying ESR2 as a mediator of E2 effect on Notch1 activation in SC.

**Fig. 3:**
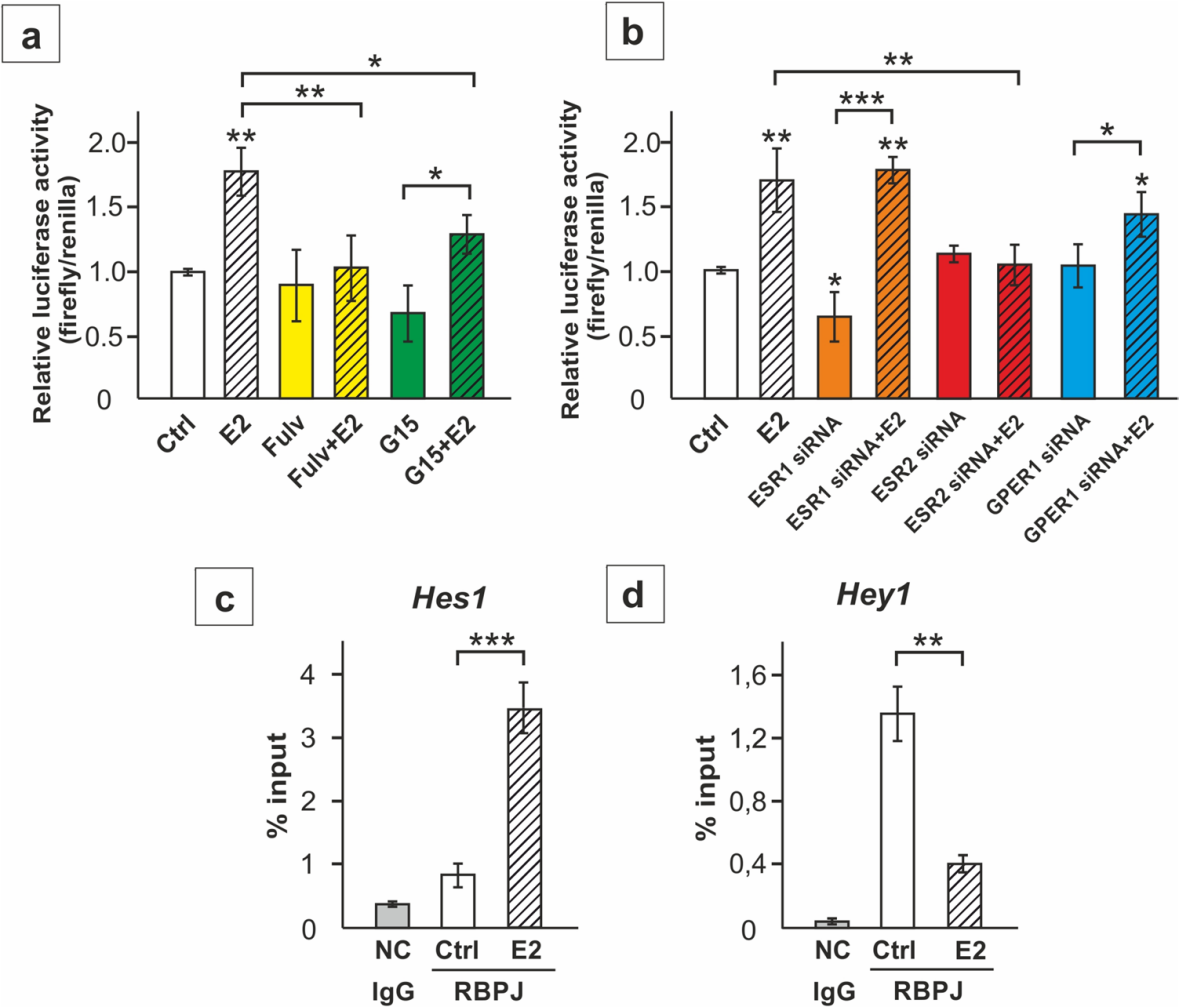
The effect of estrogen receptor silencing on RBPJ transcriptional activity (**a**-**b**) and the effect of 17β-estradiol (E2) on RBPJ interaction with *Hes1* and *Hey1* promoters (**c**-**d**) in TM4 Sertoli cells. **a** Cells were treated with 1 nM E2 and/or 1 µM fulvestrant (Fulv) or 10 nM G15, or a vehicle (Ctrl) for 24 h. **b** Cells were transfected with 2 pM of RBPJ reporter and 50 nM non-targeting siRNA (Ctrl), 50 nM ESR1 siRNA, 50 nM ESR2 siRNA, or 100 nM GPER1 siRNA, and 1 nM E2 or vehicle. Quantitative representation of firefly-derived luminescence over renilla-derived luminescence ratio (mean ± SD). **c**-**d** ChIP-qPCR analyses of the mouse *Hes1* and *Hey1* promoters showing binding of RBPJ in vehicle (Ctrl) or 17β-estradiol (E2)-treated cells. Control reactions were performed with nonspecific IgG (NC). Results are computed as percent antibody bound per input DNA, then normalized to IgG controls. The histograms are the quantitative representation of data (mean ± SD); *p < 0.05, **p < 0.01, ***p < 0.001

Increased activity of Notch pathway in response to ESR2 activation by E2 has been further confirmed based on the analysis of RBPJ transcriptional activity. RBPJ is an essential component of the Notch co-activator complex involved in canonical Notch signaling [11]. The analysis of relative luciferase activity demonstrated the induction of transcriptional RBPJ activity in TM4 cells treated with E2 (*p* < 0.01), which was inhibited by fulvestrant and *Esr2* knockdown (*p* < 0.01). After silencing of *Esr1* or *Gper1* E2-stimulated RBPJ activity persisted, indicating that these receptors play a minor role in regulating the canonical Notch pathway in SC (Fig. 3a, b).

As demonstrated using ChIP-qPCR, induction of transcriptional RBPJ activity by E2 was related to increased RBPJ binding to the promoter of Notch pathway effector gene *Hes1* (*p* < 0.001) (Fig. 3c). At the same time decreased binding of RBPJ to the promoter of another Notch-responsive gene, *Hey1* (*p* < 0.01) was detected (Fig. 3d).

**Fig. 4:**
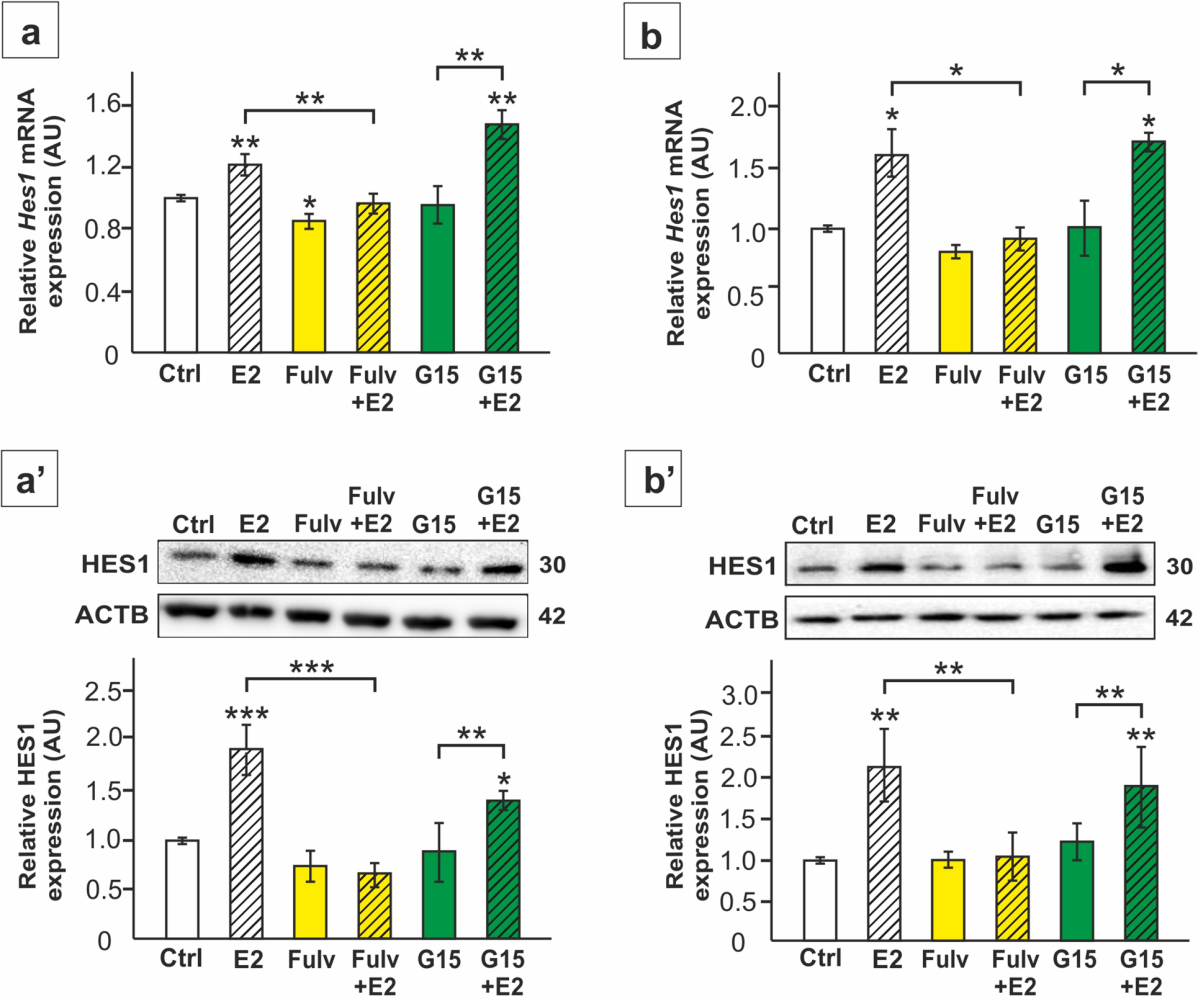
The effect of 17β-estradiol (E2) and antagonists of the estrogen receptors on *Hes1* mRNA and HES1 protein expression in TM4 Sertoli cell line (**a**-**a’**) and primary rat Sertoli cells (**b**-**b’**). Cells were treated with E2 and/or 1 µM fulvestrant (Fulv) or 10 nM G15, or a vehicle (Ctrl) for 24 h. **a**, **b** Relative expression of *Hes1* mRNA (RQ) was determined using RT-qPCR. The expression values of the individual genes were normalized to the mean expression of the reference genes (*Actb*, *Hprt1*, and *Rpl13a*) as an internal control. **a’**, **b’** Immunoblotting of HES1 protein. Protein molecular weight (in kDa) is indicated. The protein levels (normalized to ACTB) within the control group were set at 1. The histograms are the quantitative representation of data (mean ± SD); *p < 0.05, **p < 0.01, ***p < 0.001

**Fig. 5:**
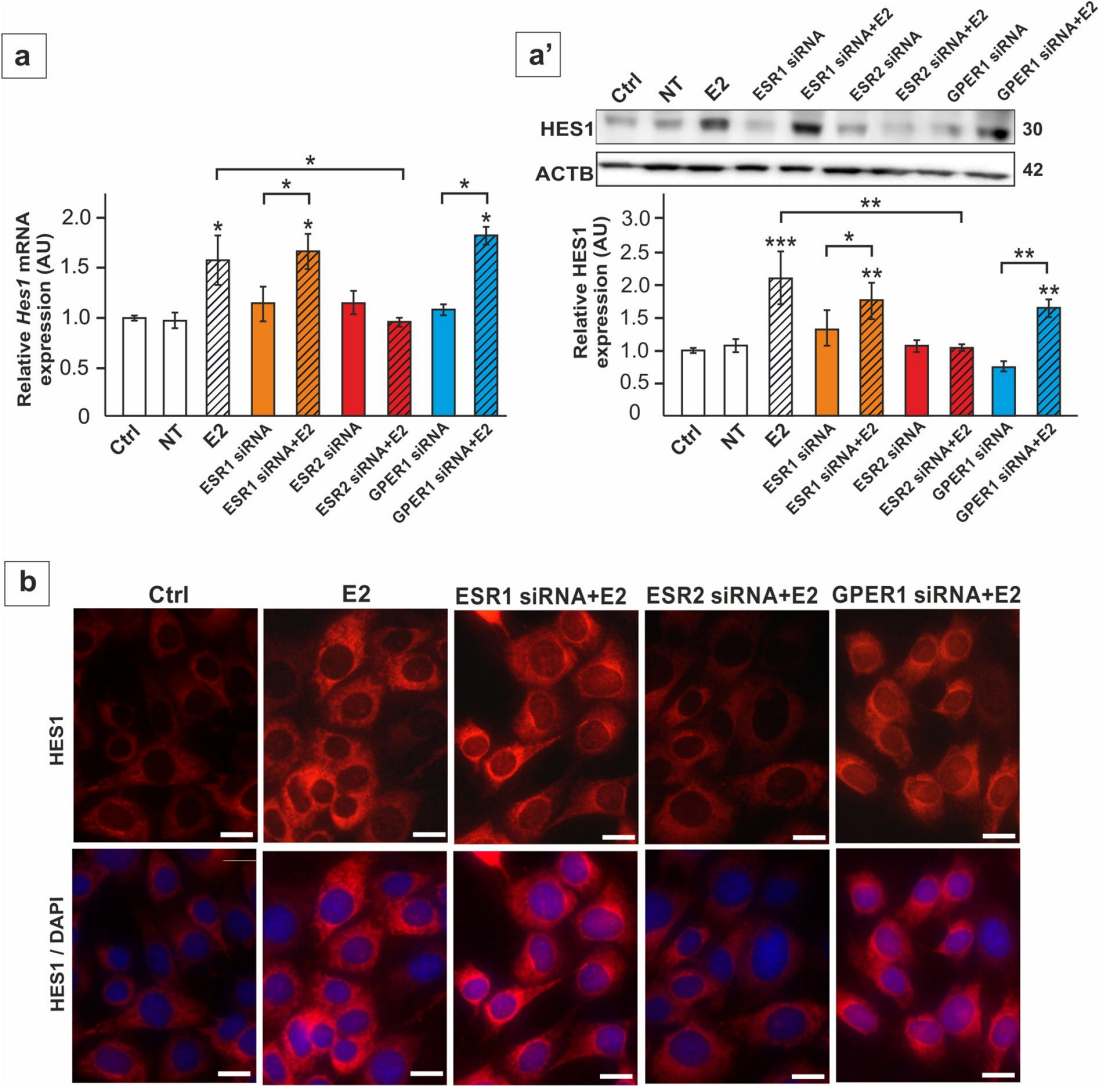
The effect of estrogen receptor silencing on *Hes1* mRNA (**a**) and HES1 protein expression (**a’**-**b**) in TM4 Sertoli cell line. Cells were treated with Lipofectamine + non-targeting siRNA (NT), Lipofectamine + 50 nM ESR1 siRNA, 50 nM ESR2 siRNA or 100 nM GPER1 siRNA and 1 nM E2 or vehicle. **a** Relative expression of *Hes1* mRNA (RQ) was determined using RT-qPCR. The expression values of the individual genes were normalized to the mean expression of the reference genes (*Actb*, *Hprt1*, and *Rpl13a*) as an internal control. **a’** Immunoblotting of HES1 protein. Protein molecular weight (in kDa) is indicated. The protein levels (normalized to ACTB) within the control group were set at 1. The histograms are the quantitative representation of data (mean ± SD); *p < 0.05, **p < 0.01, ***p < 0.001. **b** Immunofluorescence analysis of HES1 in TM4 cells. HES1 signal (red) is detected predominantly in cell cytoplasm. An increase in signal intensity is visible after E2 mainly in perinuclear region, except for the *Esr2* knockdown group. Sertoli cell nuclei were stained with DAPI (blue). Lower panel represents the merged images. Scale bar = 10 µm

Further analysis of Notch pathway effector genes in TM4 cells and primary SC revealed upregulation of *Hes1* mRNA and protein expression in response to E2 (*p* < 0.05; p < 0.01; p < 0.001) (Fig. 4a-b’, 5a,a’). Incubation of the cells with fulvestrant and *Esr2* silencing blocked this effect (*p* < 0.05; p < 0.01; p < 0.001), whereas G15 treatment and *Esr1* or *Gper1* knockdown did not prevent E2-induced *Hes1* expression (Fig. 4a-b’, 5a,a’). In agreement, immunofluorescence signal of HES1 was enhanced by E2 mainly in perinuclear region and reduced only in *Esr2* knockdown cells when compared to only E2-treated culture (Fig. 5b). These results show that changes in *Hes1* expression follow changes in Notch1 activation in response to E2-ESR2 signaling.

**Fig. 6:**
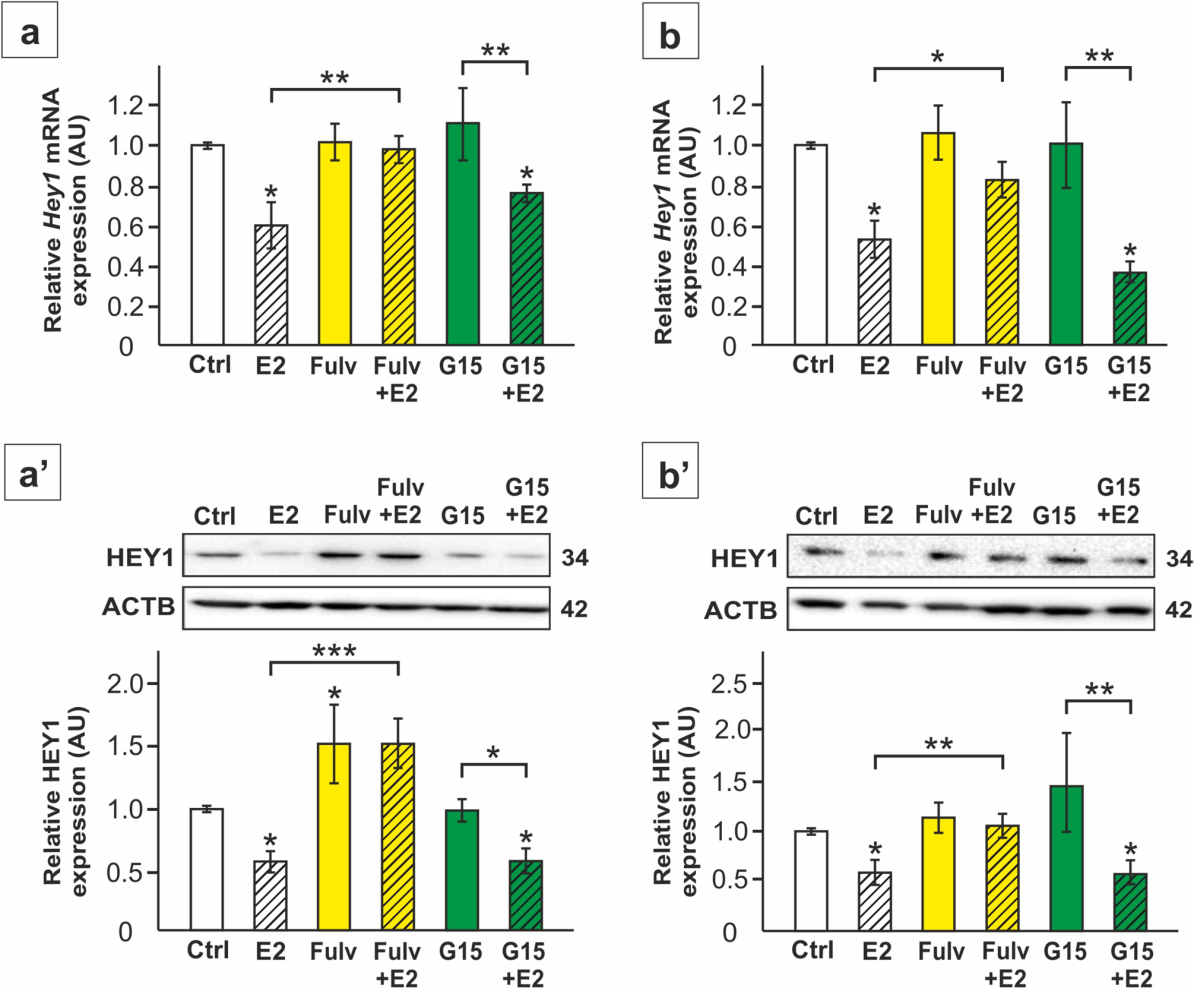
The effect of 17β-estradiol (E2) and antagonists of the estrogen receptors on *Hey1* mRNA and HEY1 protein expression in TM4 Sertoli cell line (**a**-**a’**) and primary rat Sertoli cells (**b**-**b’**). Cells were treated with 1 nM 17β-estradiol (E2) and/or 1 µM fulvestrant (Fulv) or 10 nM G15, or a vehicle for 24 h. **a**, **b** Relative expression of *Hey1* mRNA (RQ) was determined using RT-qPCR. The expression values of the individual genes were normalized to the mean expression of the reference genes (*Actb*, *Hprt1*, and *Rpl13a*) as an internal control. **a’**,**b’** Immunoblotting of HEY1 protein. Protein molecular weight (in kDa) is indicated. The protein levels (normalized to ACTB) within the control group were set at 1. The histograms are the quantitative representation of data (mean ± SD); *p < 0.05, **p < 0.01, ***p < 0.001

**Fig. 7:**
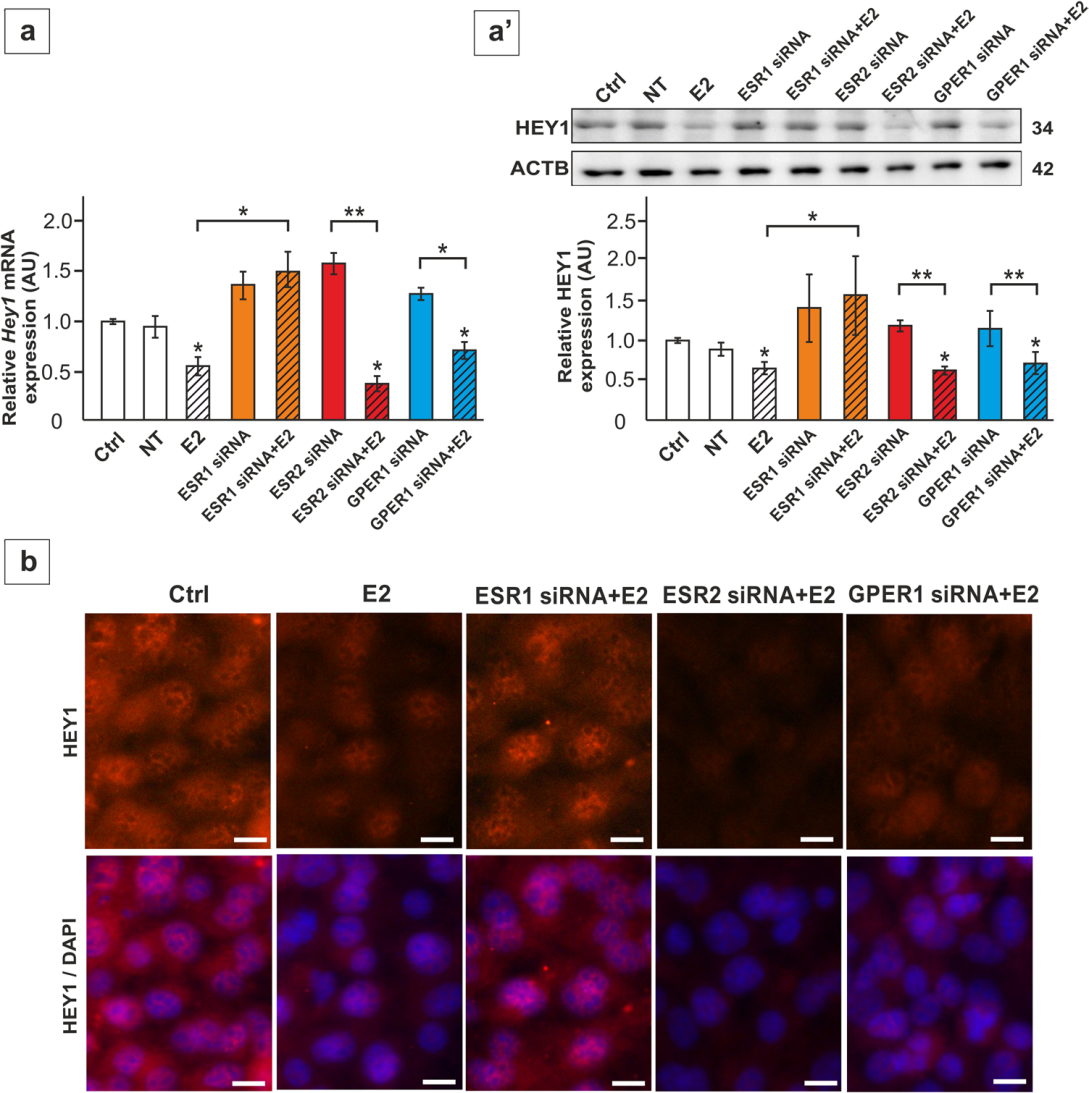
The effect of estrogen receptor silencing on *Hey1* mRNA (**a**) and HEY1 protein expression (**a’**-**b**) in TM4 Sertoli cell line. Cells were treated with Lipofectamine + non-targeting siRNA (NT), Lipofectamine + 50 nM ESR1 siRNA, 50 nM ESR2 siRNA, or 100 nM GPER1 siRNA and 1 nM E2 or vehicle. **a** Relative expression of *Hey1* mRNA (RQ) was determined using RT-qPCR. The expression values of the individual genes were normalized to the mean expression of the reference genes (*Actb*, *Hprt1*, and *Rpl13a*) as an internal control. **a’** Immunoblotting of HEY1 protein. Protein molecular weight (in kDa) is indicated. The protein levels (normalized to ACTB) within the control group were set at 1. The histograms are the quantitative representation of data (mean ± SD); *p < 0.05, **p < 0.01, ***p < 0.001. **b** Immunofluorescence analysis of HEY1 in TM4 cells. Nuclear and cytoplasmic HEY1 immunostaining (red) is detected in all experimental groups. Signal intensity is reduced after E2, except for the *Esr1* knockdown group. Sertoli cell nuclei were stained with DAPI (blue). Lower panel represents the merged images. Scale bar = 10 µm

Conversely, the expression of *Hey1* mRNA and protein was downregulated by E2 in TM4 cells and primary SC (*p* < 0.05) (Figs. 6a-b’, 7a, a’). Both, fulvestrant and silencing of *Esr1* prevented the effect of E2 on *Hey1* expression, while reduced expression was still found in the cells treated with E2 after G15 administration as well as after *Esr2* and *Gper1* knockdown (*p* < 0.05; *p* < 0.01; *p* < 0.001) (Figs. 6a-b’, 7a,a’). HEY1 immunofluorescence signal was detected in both cell cytoplasm and nuclei, and decreased markedly following E2 administration (Fig. 7b). In *Esr1* knockdown cells treated with E2, immunofluorescence intensity was maintained at the level observed in the control cells, which indicates that ESR1 is necessary for E2 mediated regulation of HES1. In contrast, *Esr2* and *Gper1* knockdown cells incubated with E2 exhibited reduced signal intensity compared to the control (Fig. 7b). Taken together, these results indicate the role of E2-ESR1 signaling in the control of HEY1 expression in SC.

## Discussion

The research presented herein has provided novel data on the role of estrogen signaling in the control of Notch pathway activity in SC. Experimental activation or inhibition of estrogen receptors performed in our study has revealed ESR2-dependent control of *Notch1* expression in SC, similarly, as previously detected in murine neural stem cells [26]. A direct regulation of Notch1 expression by E2 in SC may be postulated based on the documented presence of estrogen-response elements (ERE) in the promoter of human *NOTCH1* [27]. Further we have found that not only Notch1 expression, but also the activity of canonical Notch pathway, manifested by a higher N1ICD level present in estrogen-treated cells and increased transcriptional activity of RBPJ, is enhanced by E2-ESR2 signaling. Increased Notch1 cleavage was previously found in other cell types, such as endothelial cells, treated with E2 [28]. Of note, RBPJ-binding elements are localized in a close proximity to ERE sequences in human and rodent genomes [29]. N1ICD/RBPJ was showed to physically interact with ESR1 in breast cancer cells, forming a common complex bound to DNA, which enables ESR1-dependent activation of Notch1-mediated signaling [29]. Thus, it cannot be ruled out that such mechanisms (herein involving ESR2) are not unique for cancer cells, but are also operating in physiological conditions in SC.

Our results from ChIP-qPCR revealed enhanced binding of the RBPJ to the *Hes1* gene promoter in response to E2 followed by upregulation of *Hes1* mRNA and protein expression. The role of ESR2 in *Hes1* upregulation was also found in neuronal stem cells mentioned above [26]. Notably, ERE was identified in the promoter of *HES1* gene and both ESR1 and ESR2 were found to control *HES1* expression in human cancer cells [30, 31]. A slightly different pattern of the response is observed in the SC analyzed in our study, where only ESR2 has been involved in upregulating Notch pathway activity and HES1 expression, while binding of E2 to ESR1 reduced RBPJ interaction with *Hey1* promoter and decreased the expression of the HEY1. This clearly points to a divergent roles of ESR1 and ESR2 in SC in the control of Notch pathway activation. Distinct roles of ESR1 and ESR2 in regulating cellular process were documented previously regarding their effects on SC growth and maturation during postnatal testis development or the expression of the ligands for Notch receptors [9, 23, 32].

Previous findings revealed that a specific concentration of NICD is necessary to initiate the transcriptional activation of particular target genes [33, 34]. Therefore, transcriptional responses of *Hes1* and *Hey1* to Notch pathway activation in SC may differ due to specific response thresholds of these genes [35]. It is also worth noting that HES1, forming a heterodimer with HEYL, binds to the *Hey1* promoter, which implies that *Hey1* transcription may be directly modulated by HES1 [24]. In the context of *Hey1* downregulation by E2 in the present study, the possibility that *Hey1* is controlled by estrogens independently on canonical Notch pathway should be also considered. Noteworthy, Zhang et al. [36] demonstrated that in mouse uterine cells RBPJ acts both directly, via a Notch-dependent mechanism and indirectly, via physically interacting with ESR1 in a Notch-independent manner. Earlier studies in several normal and cancerous cell types reported diverse response of *Hey1* to estrogen signaling manipulation, which indicates clearly cell context-dependent regulation of this gene [37–39].

Differential modulation of Notch downstream pathways in SC by ESR1 and ESR2 suggests that ESR1 (by downregulating *Hey1*) may serve as a counter-regulatory mechanism to fine-tune Notch-mediated effects. Thus, ESR1 signaling may dampen or modulate some aspects of Notch pathway possibly to balance cellular responses. However, it should be taken into account that the expression level of ESR1 is low in comparison to ESR2 in postproliferative rodent SC [9, 23]. Therefore, E2-ESR2-HES1 pathway seems to play a dominant role in rodent SC, whereas physiological significance of E2-ESR1-HEY1 might be limited.

Although the involvement of GPER1 in the control of Notch1-HES1 signaling was reported in breast cancer cell lines [40], we have found no direct role of GPER1 in regulating the activity of Notch pathway in SC. It is worth mentioning, however, that E2-GPER1 signaling has a significant impact on SC expression of Delta-like 4, a canonical ligand of Notch receptors [23]. Thus, the effect of E2-GPER1 pathway on Notch signaling activity in seminiferous epithelium cannot be ruled out and require closer examination.

Finally, both estrogen and the Notch signaling are involved in regulating several common cellular functions in SC, such as blood-testis barrier (e.g. the expression of claudin-5) or apoptosis-related proteins (e.g. the expression of FasL) [13, 16, 41, 42], which may indicate the specific aspects of SC physiology where the relationship between estrogens and Notch pathway plays a significant role. Estrogen-Notch interplay may be also worth attention in the context of the well-documented disturbing effects of xenoestrogens on the function of the male gonad [43, 44]. Endocrine disruptors of estrogenic mode of action, such as bisphenol A or zearalenone were shown to dysregulate Notch pathway activity in different cell populations of developing and adult testis, accompanied by altered cell differentiation, steroidogenesis, and the stem cell niche [45–47].

## Conclusion

The data presented in this study reveal a new mechanism by which estrogens may influence the homeostasis of SC. Our findings demonstrate that the estrogen receptors have distinct roles in regulating Notch signaling in SC. The activation of ESR2 is a key driver in stimulation of canonical Notch1-RBPJ/HES1 pathway activity by estrogens. On the other hand, ESR1 may be involved in decreasing HEY1 expression by E2 via the RBPJ-dependent, but likely Notch1-independent mechanism, possibly contributing to a balanced cellular outcome. Therefore, it is worthwhile to undertake further research to elucidate how the crosstalk between estrogens and the Notch pathway may regulate SC physiology and to explain the significance of this regulation in seminiferous epithelium and, consequently, the course of spermatogenesis.

## Supplementary Information

Below is the link to the electronic supplementary material.

ESM 1 (DOCX 16.8 KB)

## Data Availability

The data that support the findings of this study are available from the corresponding author upon reasonable request.
