# Supplementary material for: Role of Estrogen Signaling in Notch Pathway Activation in Sertoli Cells
Source: Reprod Sci. 2025 Jun 12;32(7):2307–18. doi: 10.1007/s43032-025-01901-y (PMC12270961; doi:10.1007/s43032-025-01901-y)
Supplement: Supplementary file 1 — (DOCX 16.8 KB) [file 43032_2025_1901_MOESM1_ESM.docx]

**Supplementary Table S1.** Sequences of forward and reverse primers

| **Gene** | **Forward primer** | **Reverse primer** | **Annealing temperature [°C]** |
| --- | --- | --- | --- |
| **Rat** |  |  |  |
| Actb | CACACTGTGCCCATCTATGA | CCGATAGTGATGACCTGACG | 58 |
| Hes1 | GGCAGGCGCACCCCGCCTTG | GCAGCCAGGCTGGAGAGGCT | 62 |
| Hey1 | AAAGACGGAGAGGCATCATCG | GCAGTGTGCAGCATTTTCAGG | 55 |
| Hprt1 | GACTTTGCTTTCCTTGGTCA | AGTCAAGGGCATATCCAACA | 58 |
| Notch1 | GCAGCCACAGAACTTACAAATCCAG | TAAATGCCTCTGGAATGTGGGTGAT | 56 |
| Rpl13a | GTGAGGGCATCAACATTTCT | CATCCGCTTTTTCTTGTCAT | 58 |
| **Mouse** |  |  |  |
| Actb | AAGAGCTATGAGCTGCCTGA | TACGGATGTCAACGTCACAC | 58 |
| Hes1 | ACCTTCCAGTGGCTCCTC | TTTAGTGTCCGTCAGAAGAGAG | 52 |
| Hey1 | GCCGAAGTTGCCCGTTATCTG | GCCGAAGTTGCCCGTTATCTG | 53 |
| Hprt1 | GCTGACCTGCTGGATTACAT | TTGGGGCTGTACTGCTTAAC | 58 |
| Notch1 | GATGCCACCTGAACAACTGC | TGACAACAGCAACAGCAAGG | 62 |
| Rpl13a | ATGACAAGAAAAAGCGGATG | CTTTTCTGCCTGTTTCCGTA | 58 |

**Supplementary Table S2.** Details of primary antibodies used for immunoblotting and immunofluorescence

| **Antibody** | **Host species** | **Vendor** | **Cat. number** | **Dilution** |
| --- | --- | --- | --- | --- |
| Anti‐ACTB | Mouse | Sigma‐Aldrich | A2228 | 1:3000 (IB) |
| Anti-HES1 | Rabbit | Thermo Fischer | PA5-28802 | 1:1000 (IB); 1:100 (IF) |
| Anti‐HEY1 | Rabbit | Thermo Fischer | PA5‐40553 | 1:2000 (IB); 1:100 (IF) |
| Anti‐N1ICD | Rabbit | Abcam | ab8925 | 1:1000 (IB); 1:200 (IF) |

IB – immunoblotting; IF – immunofluorescence
